# ASO Author Reflections: It Takes Two to SAMBA—Toward Standardized Robotic Parenchymal Transection with Sealing Devices

**DOI:** 10.1245/s10434-026-19467-7

**Published:** 2026-03-10

**Authors:** Emrullah Birgin, Nuh N. Rahbari

**Affiliations:** Department of General and Visceral Surgery, Ulm University Hospital, Ulm, Germany

## Past

Minimally invasive liver surgery has evolved from laparoscopic to robotic hepatectomy, with the robotic approach providing enhanced visualization, dexterity, and precision.^1^ Laparoscopic parenchymal transection techniques have become standardized over recent decades, but the range of instruments available for parenchymal transection on the robotic platform remains limited.^2^ As a result, many hepatobiliary centers continue to rely on hybrid parenchymal transection techniques. We have previously described a standardized parenchymal transection technique using monopolar scissors and bipolar forceps.^3^ However, comprehensive procedural descriptions detailing effective and reproducible application of robotic sealing devices—such as the SynchroSeal and Vessel Sealer Extend—are still lacking.

## Present

The Sealer and Moisture-Based Approach (SAMBA) hepatectomy was developed to standardize robotic parenchymal transection using the SynchroSeal and Vessel Sealer Extend.^4^ The technique structures transection into four reproducible phases: capsular incision, seal–irrigation–release sequence, crush-clamping, and targeted dissection. A central principle of SAMBA is synchronization. The deliberate coordination between console surgeon and bedside assistant—alternating sealing, saline irrigation, and controlled release—reduces carbonization, minimizes tissue adherence, and preserves visualization with steady progression through the resection plane. By integrating moisture to energy delivery, SAMBA transforms robotic sealing devices into precise instruments for dissection and transection. In a consecutive series of 72 robotic hepatectomies including predominantly anatomical resections (63%), the technique demonstrated favorable operative times, limited blood loss, low bile leak rates, and no 90-day mortality. These findings support the feasibility and safety of a standardized, energy-based, purely robotic approach across a broad spectrum of procedural complexity.

## Future

Standardization represents the next chapter in the maturation of robotic hepatectomy, with the potential to reduce variability in outcomes and facilitate structured training.^5^ Future comparative studies in robotic liver surgery should therefore rely on clearly defined and standardized techniques. Prospective clinical trials are required to compare the SAMBA technique with other parenchymal transection techniques. As robotic liver surgery continues to expand worldwide, innovation alone will not suffice—systematic standardization may ultimately define its trajectory.
